# Effects of 2.45-GHz Electromagnetic Fields with a Wide Range of SARs on Micronucleus Formation in CHO-K1 Cells

**DOI:** 10.1100/tsw.2004.176

**Published:** 2004-10-20

**Authors:** S. Koyama, Y. Isozumi, Y. Suzuki, M. Taki, J. Miyakoshi

**Affiliations:** ^1^ Department of Radiological Technology, School of Health Sciences, Faculty of Medicine, Hirosaki University, 66-1 Hon-cho, Hirosaki, 036-8564, Japan; ^2^ Department of Interdisciplinary Environment, Graduate School of Human and Environmental Studies, Kyoto University, Yoshida-Konoe-cho, Sakyo-ku, Kyoto 606-8501, Japan; ^3^ Department of Electrical Engineering, Graduate School of Engineering, Tokyo Metropolitan University, 1-1 Minami Ohsawa, Hachioji, Tokyo 192-0397, Japan

**Keywords:** high-frequency electromagnetic fields, HFEMF, micronucleus, MN, specific absorption rates, SARs, combination effects, bleomycin

## Abstract

There has been considerable discussion about the influence of high-frequency electromagnetic fields (HFEMF) on the human body. In particular, HFEMF used for mobile phones may be of great concern for human health. In order to investigate the properties of HFEMF, we have examined the effects of 2.45-GHz EMF on micronucleus (MN) formation in Chinese hamster ovary (CHO)-K1 cells. MN formation is induced by chromosomal breakage or inhibition of spindles during cell division and leads to cell damage. We also examined the influence of heat on MN formation, since HFEMF exposure causes a rise in temperature. CHO-K1 cells were exposed to HFEMF for 2 h at average specific absorption rates (SARs) of 5, 10, 20, 50, 100, and 200 W/kg, and the effects on these cells were compared with those in sham-exposed control cells. The cells were also treated with bleomycin alone as a positive control or with combined treatment of HFEMF exposure and bleomycin. Heat treatment was performed at temperatures of 37, 38, 39, 40, 41, and 42°C.The MN frequency in cells exposed to HFEMF at a SAR of lower than 50 W/kg did not differ from the sham-exposed controls, while those at SARs of 100 and 200 W/kg were significantly higher when compared with the sham-exposed controls. There was no apparent combined effect of HFEMF exposure and bleomycin treatment. On heat treatment at temperatures from 38–42°C, the MN frequency increased in a temperature-dependent manner. We also showed that an increase in SAR causes a rise in temperature and this may be connected to the increase in MN formation generated by exposure to HFEMF.

### INTRODUCTION

In the modern era, people are exposed to many kinds of electromagnetic fields (EMF) and exposure occurs almost all day long. The use of domestic electrical appliances has increased and mobile telephones are used extensively all over the world. Several epidemiological studies have indicated that exposure to EMF may be associated with an increased risk of childhood leukemia and other cancers[1,2,3,4], and this has caused great interest in the field. Some positive data have indicated a correlation between exposure to environmental low-frequency EMF and DNA damage or chromosomal aberrations, which may induce certain kinds of disease[5,6,7]. However, it has also been strongly suggested that no such relationship exists between EMF exposure and carcinogenesis, or that, if there is a relationship, it is only a weak one[8]. In addition to the conflicting and contradictory views on extremely low-frequency EMF, there is also controversy surrounding the effects of high-frequency electromagnetic fields (HFEMF)[9,10]. HFEMF are particularly used in mobile communication and, hence, there is considerable interest in the possible effects of HFEMF on human health.

The potential hazards of exposure to HFEMF have been addressed in many epidemiological studies[11,12,13], however, it remains unclear whether there is an association between exposure to HFEMF and human health or development. Some reports have suggested that effects caused by increased temperature cannot be ruled out as an explanation of the relationship between HFEMF and adverse outcomes[14,15]. On the other hand, numerous reports have shown that HFEMF exert significant effects under isothermal conditions[16,17,18].

Considering these previous data, we have examined the frequency of micronucleus (MN) formation in Chinese hamster ovary (CHO) K-1 cells following HFEMF exposure. MN are derived from clastogenic activity arising from chromosomal fragments that are not incorporated into daughter nuclei at mitosis. This occurs because of the lack of a kinetochore. MN are also derived from an aneugenic process when the whole chromosome is not correctly integrated into the two daughter nuclei at the time of cell division[19,20,21,22]. MN formation was investigated under the following conditions: on exposure to HFEMF at a range of average specific absorption rates (SARs) from 5–200 W/kg with or without bleomycin and on heat treatment with or without bleomycin. In addition, MN formation on sham exposure (negative control) and with bleomycin treatment alone (positive control) was examined at 37°C. All treatments were performed for 2 h.

### MATERIALS AND METHODS

#### Cells and Culture Conditions

CHO-K1 cells were obtained from the Japanese Cancer Research Bank, Tokyo. Cells were maintained in Ham’s F-12 medium (Nikken) supplemented with 10% fetal bovine serum (Gibco, BRL) at 37°C in 95% air and 5% $CO_2$. Cells suspensions at a concentration of $1 \times 10^6$ cell/ml in a volume of 20.1 ml were seeded on a section of a special culture dish designed to allow exposure to an EMF. Following incubation for 23 h, the cells were treated as described below.

#### Exposure System

##### *Waveguide Applicator*

Fig. 1(a) is a photograph of the exposure apparatus and a schematic view of the applicator by which the cells are exposed to HFEMF of 2.45-GHz sinusoidal continuous waves (CW) is shown in Fig. 1(b). The applicator is based on a rectangular waveguide, with the size of the waveguide determined to be 110 mm (width) × 55 mm (height) × 310 mm (depth). The electromagnetic waves are propagated along the

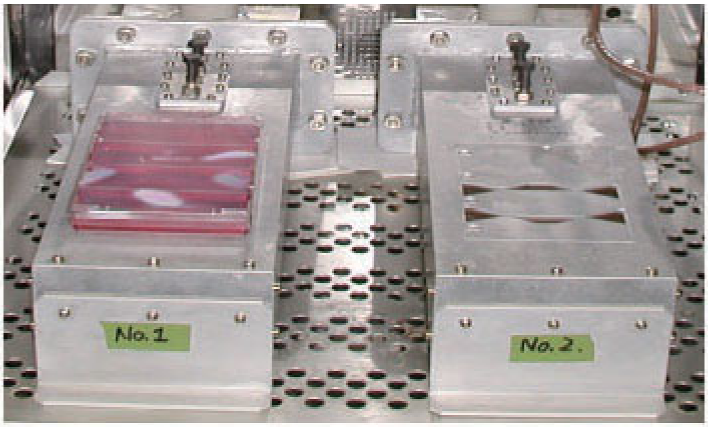

**FIGURE 1(a).** A photograph of the exposure apparatus.

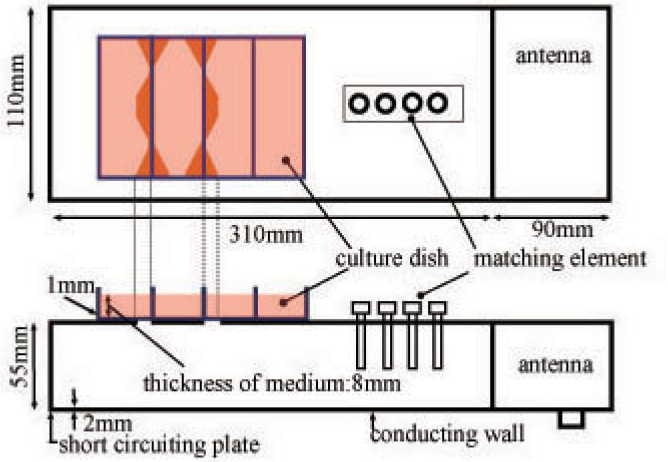

**FIGURE 1(b)**

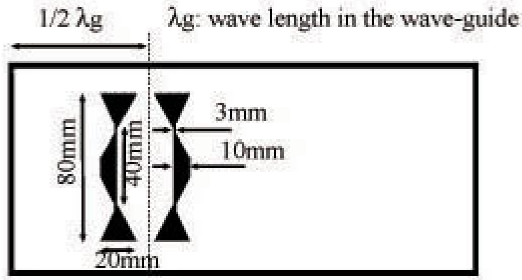

**FIGURE 1(c)**

**FIGURE 1(b) and (c).** Schematic view of the applicator.

waveguide in $TE_{10}$ mode. One end of the applicator is terminated with a short-circuiting plate to generate standing waves in the waveguide.

A culture dish is placed on two slits which are bored into the wider wall of the waveguide, and cells in the culture dish are exposed to the HFEMF through these slits. The two slits are pinch shaped (Fig. 1[c]), which enhances the coupling of the EMF to increase the effective exposure area. The culture dish used is of a rectangular shape and is divided into four compartments, as shown in Fig. 1(b). The distribution of the electric field in the medium depends on the thickness of the medium. Therefore, it is preferable to choose a thickness that maximizes the SAR at the bottom of the medium where the cells are present. Numerical analysis indicates that the appropriate thickness is 8 mm, which corresponds to a half wavelength in the medium.

The SAR distribution in the exposure apparatus was calculated using the Finite Difference Time Domain (FDTD) method. Fig. 2(b) shows the SAR at the bottom of the medium along x-axis in Fig. 2(a) when the incident electric power is 1 W. Each compartment has a different value of SAR, as shown in Fig. 2(b). The maximum SAR appears at the bottom of position P in Fig. 2(a) in compartment 3. The maximum SARs value in the compartment 2 and 4 are 80 and 85% of the maximum SAR value in the compartment 3. The minimum SAR in the compartment 1 is 1% of the maximum value at the bottom of position P.

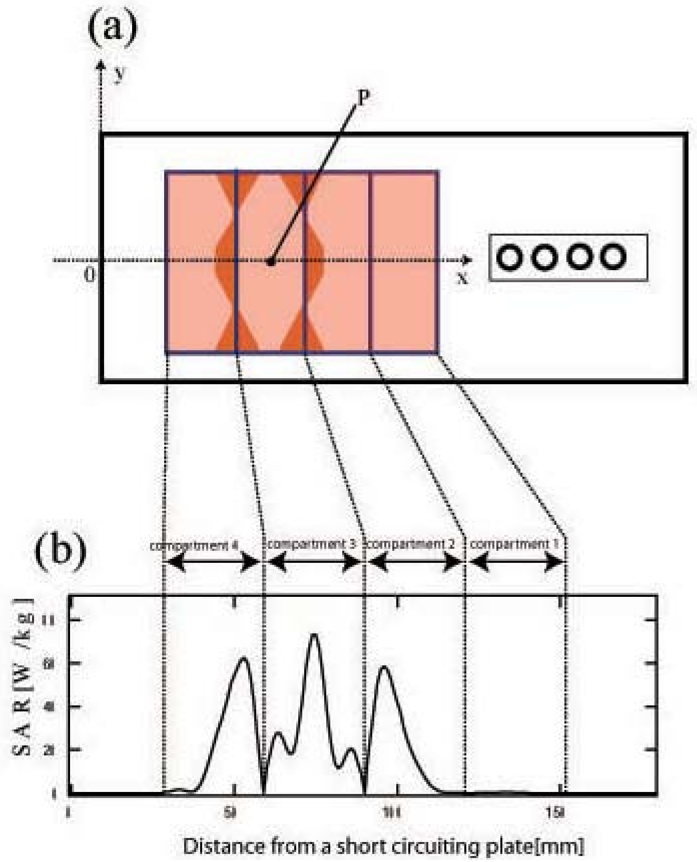

**FIGURE 2**. SARs distribution in the exposure setup, obtained from calculations using the FDTD method.

The advantages of the waveguide applicator are as follows: (1) a high SAR is achieved at the cell location with strong EMF coupling, (2) temperature control is straightforward because the bottom of the culture dish makes contact with the metal wall of the waveguide, (3) the compact design allows the use of ordinary incubators to facilitate suitable cell culture conditions, and (4) the applicator is simple and easy to set up the experiment, due to the location of the culture dish, which is merely required to be placed on the slits that are outside the waveguide.

A schematic view of the exposure system is shown in Fig. 3. The applicator is mounted in an incubator. Continuous wave signal generated by a signal generator (Agilent E4438) is amplified by a power amplifier (R&K A0825-5050-R) and the amplified wave signal is directed into the exposure applicator. Impedance matching is established by adjusting matching elements. The incident and reflected electromagnetic powers are monitored by power sensors and the incident power is precisely controlled by the signal generator.

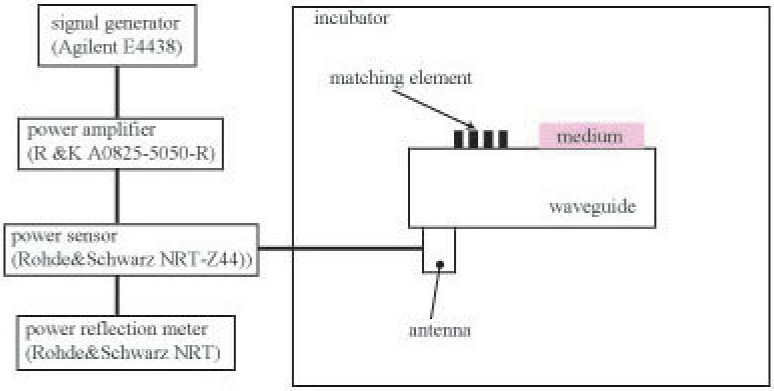

**FIGURE 3**. Schematic view of the exposure system.

The SAR is determined experimentally from the increase in the medium temperature during exposure over a short time. The maximum SAR value obtained by measurement is about 73 W/kg at the bottom of position P for an incident power of 1 W. The temperature was measured by a DASY 3 system with a thermistor probe and the SAR measurement showed good agreement with the result of a numerical calculation.

#### *Characteristic of Temperature Elevations*

Exposure to HFEMF causes temperature elevation in the medium. The temperature in the medium during exposure is measured by a fluoroptic temperature probe (Luxtron model 790). The temperature reaches a stationary value in about 2000 sec. Fig. 4 shows the temperature of the medium after 2000 sec from the onset of the exposure at the bottom of position P in Fig. 2(a). The initial value of the temperature is 37°C. The temperature evaluation is approximately proportional to the SAR value.

The temperature elevation is not homogeneous. Fig. 5 shows the temperature distribution at the bottom obtained by a numerical calculation. The calculation assumes the maximum SAR of 50 W/kg at the bottom of position P.

### HFEMF Exposure With or Without Bleomycin

After incubation for 23 h, the medium was changed, either to similar fresh medium or to a new medium containing bleomycin at a final concentration of 10 µg/ml. In each case, a further 1-h incubation was then performed. Cells treated with or without bleomycin were then washed three times with PBS, cultured in new medium, and then exposed to a HFEMF of SAR 5, 10, 20, 50, 100, or 200 W/kg for 2 h.

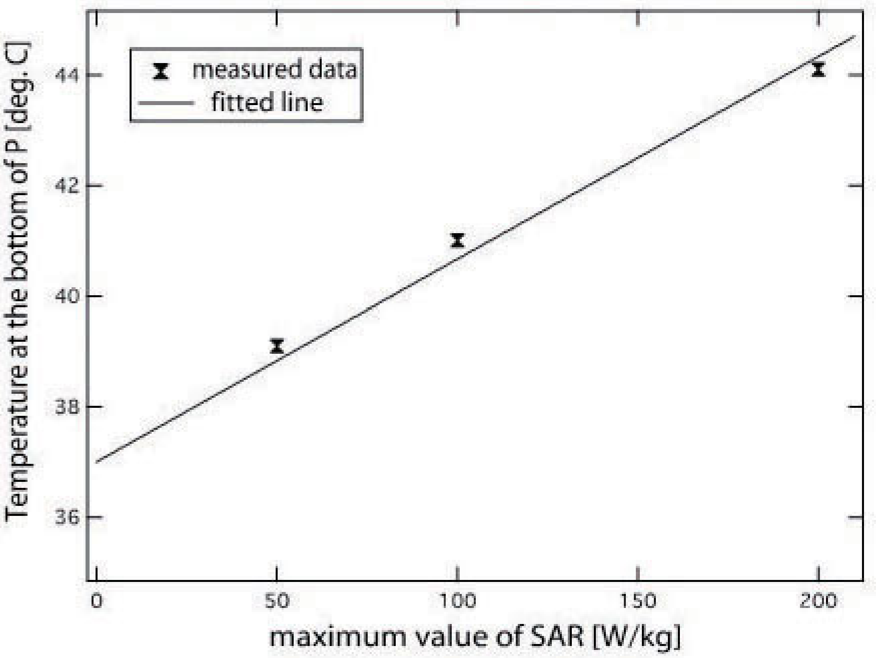

**FIGURE 4.** A graph of the temperature rising in the medium after 2000 sec from the onset of the exposure at the bottom of position P in Fig. 2(a).

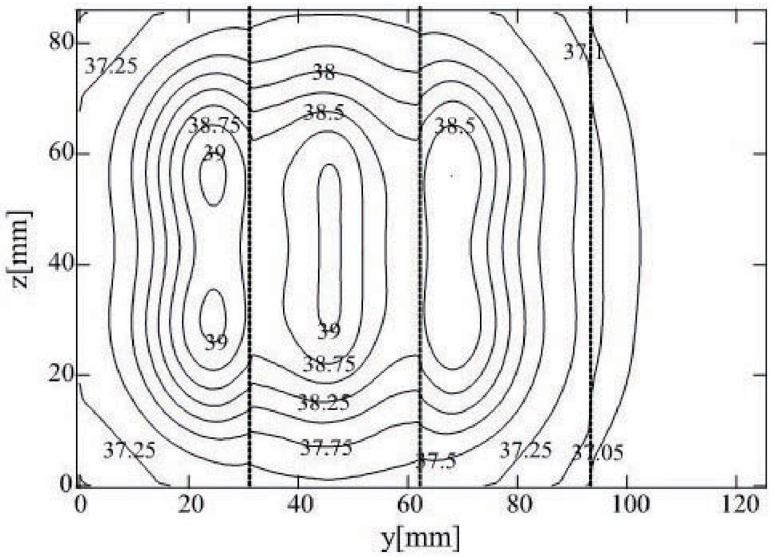

**FIGURE 5**. The temperature distribution at the bottom obtained a numerical calculation.

### Heat Treatment

After treatment with or without bleomycin, the cells were washed three times with PBS, cultured in new medium, and then incubated at 38, 39, 40, 41, or 42°C for 2 h.

### Sham Exposure and Bleomycin Treatment

A sham-exposure experiment was performed as a negative control. Cells were treated in the same way to those that underwent HFEMF exposure, except that HFEMF exposure itself was omitted. Instead, the cells were incubated for 2 h in a conventional incubator. In addition, the cells were treated with bleomycin alone as a positive control, using the same conditions as those used for sham exposure.

### Inhibition of Cytokinesis for Detection of Binucleated Cells

After each 2-h treatment, the cells were trypsinized and seeded in 10-cm culture dishes in medium containing cytochalasin B at a final concentration of 3 µg/ml for 18 h, in order to prevent cell division.

### Slide Preparation

The cells were collected at a concentration of $2.7 \times 10^4$ cells/ml and samples of 0.2 ml were centrifuged onto slides using a Cytospin centrifuge (Shandon Southern Ltd.) at $100 \times g$ for 5 min. The cells were then fixed with 80% cold ethanol for 30 min. The slides were washed gently with PBS and soaked in new PBS for 5 min to completely remove the ethanol.

### Scoring Procedure

The cells on the slides were stained with 20 µl of propidium iodide (PI) diluted in glycerol (to a concentration of 0.2 µg/ml) and kept in the dark until counting was performed. A total of 1000 binucleated cells were scored for evaluation of the frequency of induction of MN using fluorescence microscopy (Olympus). Cells were counted as having MN formation when they contained at least one MN. The procedure was performed in a double-blind manner and followed the method described by Countryman and Heddle[22]. Statistical analysis of the data in the control and experimental groups was conducted using ANOVA (analysis of variance) followed by Fisher's Protected Least Significant Difference (PLSD) test.

## RESULTS

### Formation of MN

The formation of MN is shown in Fig. 6. Fig. 6A shows a binucleated cell without MN and Fig. 6B shows a binucleated cell with MN.

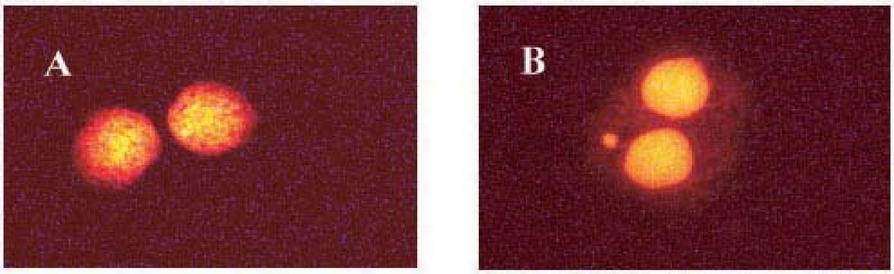

**FIGURE 6**. (A) Binucleated cell without MN; (B) Binucleated cell with MN.

### Effect of HFEMF Exposure on MN Formation

The effect on MN formation of exposure to HFEMF is shown in Fig. 7. There was no increase in MN formation in cells exposed to HFEMF at SARs from 5–50 W/kg. However, the frequency of MN formation in cells exposed to HFEMF at SARs of 100 and 200 W/kg were statistically significantly different to that observed following sham exposure.

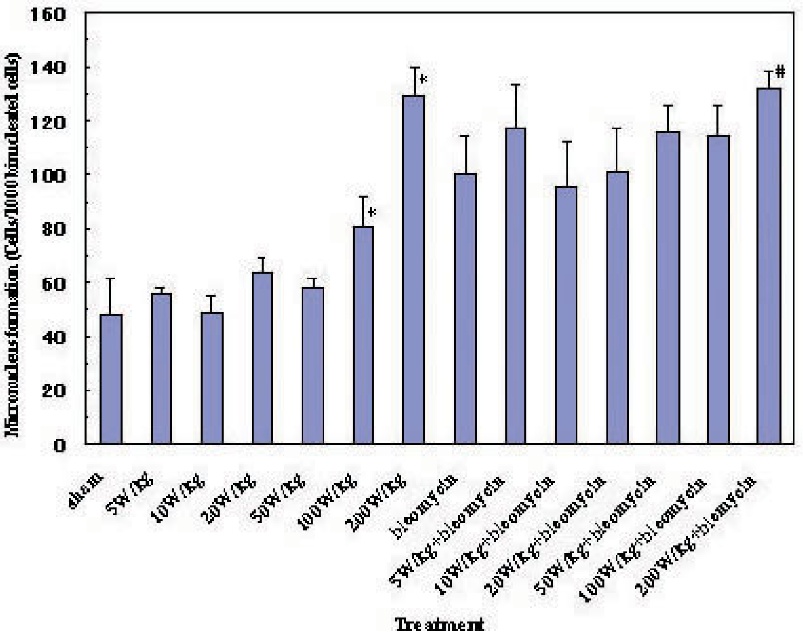

**FIGURE 7**. The frequency of MN formation in CHO-K1 cells exposed to HFEMF with or without bleomycin, compared to that following sham exposure. The columns represent means and the bars show standard deviations, based on three experiments. An asterisk indicates a statistically significant difference between sham exposure and HFEMF exposure ($p < 0.01$). A sharp indicates a statistically significant difference between treatment with bleomycin alone and combined treatment with HFEMF exposure and bleomycin ($p < 0.01$).

### Combined Effect of Bleomycin and HFEMF Exposure on MN Formation

The frequency of MN formation following treatment with bleomycin alone was statistically higher compared with the sham-exposed control (Fig. 7). Although there was no increase in MN formation in cells treated with a combination of bleomycin and HFEMF exposure at SARs from 5–100 W/kg, there was a statistically significant difference in MN formation between treatment with bleomycin alone and combined treatment with bleomycin and HFEMF exposure at a SAR of 200 W/kg.

### Effect of Heat on MN Formation

The effects of heat and change of temperature on MN formation are shown in Fig. 8. The frequency of MN formation with heat treatment increased in a temperature-dependent manner. However, there was no difference between bleomycin-treated cells and cells treated with a combination of heat and bleomycin for temperatures from 38–41°C. MN formation in cells that underwent combined treatment with heat and bleomycin at 42°C was statistically different to that in cells treated with bleomycin alone, but there was no difference in MN formation compared to that in cells treated with heat only at 42°C.

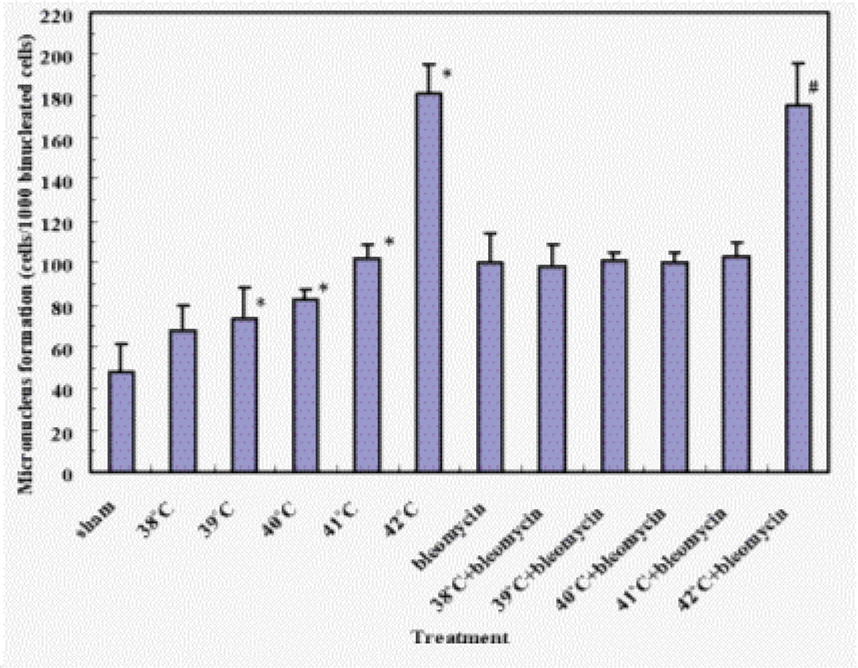

**FIGURE 8.** The effect of temperature on MN formation. The columns represent means and the bars show standard deviations, based on three experiments. An asterisk indicates a statistically significant difference between heat treatment alone and combined treatment with heat and bleomycin ($p < 0.01$). A sharp indicates a statistically significant difference between treatment with bleomycin alone and combined treatment with heat and bleomycin ($p < 0.01$).

### Relationship Between the Effect of HFEMF Exposure and Temperature

The relationship between the effect on MN formation of exposure to HFEMF and temperature is shown in Fig. 9. Columns indicate the frequency of MN formation and the line graph indicates the change of temperature generated by exposure to the HFEMF. Temperatures higher than 39°C caused a statistically significant increase in MN frequency, similar to that shown in Fig. 8. The frequency of MN formation increased in a temperature-dependent manner as the temperature was raised. A correlation coefficient of 0.978239 was obtained between the frequency of MN formation and the temperature.

### DISCUSSION

In a previous study on the effects of HFEMF on MN formation in CHO-K1 cells, we showed that exposure to HFEMF at a SAR lower than 50 W/kg did not induce MN formation, but that a statistically significant increase in MN formation at SARs of 100 and 78 W/kg did occur[23]. Moreover, previous work has shown that the MN frequency in cells exposed to HFEMF after bleomycin treatment increases in a statistically significant manner at a SAR higher than 78 W/kg, compared with bleomycin treatment alone. In the present study, there was also no difference in MN formation in CHO-K1 cells exposed to HFEMF at a SAR lower than 50 W/kg, but there were statistically significant increases in MN frequency at SARs of 100 and 200 W/kg, compared with the sham-exposed control (Fig. 7). However, we failed to detect an increase in MN formation in cells exposed to HFEMF at a SAR of 100 W/kg after bleomycin treatment, compared with bleomycin treatment alone, and combined effects of bleomycin and HFEMF exposure at SARs lower than 100 W/kg were not detected. Although we detected enhancement of MN

formation with combined treatment of HFEMF exposure at a SAR of 200 W/kg and bleomycin, an appreciable rise in temperature occurs under such conditions (Fig. 8 and 9). The MN frequency following

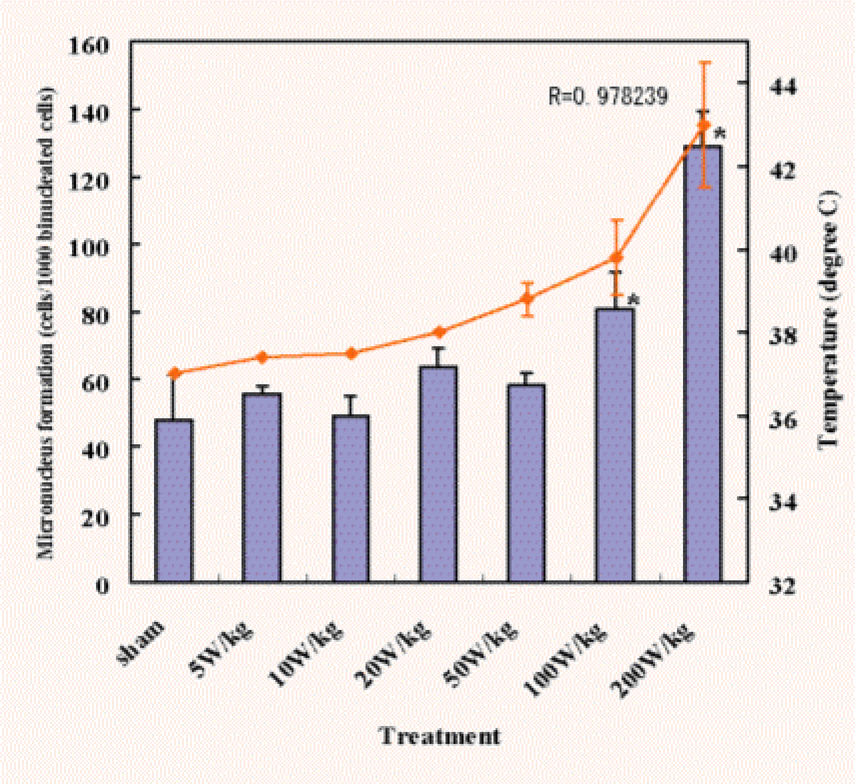

**FIGURE 9**. The relationship between the effect of exposure to HFEMF and temperature. Columns indicate the means of MN frequency and the line graph indicates the change in temperature generated by exposure to HFEMF. The bars represent standard deviations from three experiments. There were no temperature changes on sham exposure or HFEMF exposure at SARs of 5, 10, 20, and 50 W/kg. An asterisk indicates a statistically significant difference between sham exposure and HFEMF exposure ($p < 0.01$). R represents the correlation coefficient between the frequency of MN formation and temperature.

combined treatment of HFEMF exposure at a SAR of 200 W/kg and bleomycin did not differ from that with HFEMF exposure at a SAR of 200 W/kg alone. This implies that HFEMF exposure at a SAR of 200 W/kg does not enhance the effect of bleomycin. This result is in contrast to many papers that have described the adverse effects of HFEMF exposure. For example, Maes et al.[24,25] have shown a highly reproducible synergistic effect using 954 or 935.2 MHz microwaves and mitomycin C, an agent that chemically damages DNA. However, we were unable to find such synergistic effects for HFEMF exposure and bleomycin.

In the present study, we were able to show that MN formation increased in a temperature-dependent manner (Fig. 8), in agreement with several previous reports, which have also indicated that a rise in temperature can induce MN formation[26,27,28]. Other studies have indicated that significant effects on various cellular activities are obtained on exposure to radiofrequency (RF) radiation or microwaves under isothermal conditions. Hence, Cleary et al.[29] provided support for the hypothesis that RF radiation might affect membrane signal transduction in the absence of heating, resulting in altered cell proliferation, and Repacholi et al.[30] have reported that long-term intermittent exposure to RF fields increases the probability that mice carrying a lymphomagenic oncogene will develop lymphomas. The potential hazards of exposure to HFEMF under nonthermal condition have also been discussed[31]. However, in the present study, the increase in MN formation following exposure to HFEMF seems to depend on temperature rise, based on the correlation coefficient (R = 0.978239) for the data in Fig. 9. This result is supported by a literature report based on use of a pulsed electromagnetic field[32].

Lai and Singh[33,34,35] have shown DNA strand breakage in rat brain cells after exposure to EMF and genetic effects such as MN induction and DNA damage following HFEMF exposure have been reported in several different papers[36,37,38]. However, Vijayalaxmi et al.[39,40] have suggested that HFEMF exposure alone has no genotoxic effects on cells or that the relationship between RF radiation and cancer is weak to nonexistent, hence there continues to be contradictory information regarding the relationship of HFEMF exposure and human health. Although more research is required, the present study has shown that MN formation on HFEMF exposure at SARs from 5–200 W/kg is connected to the temperature rise caused by HFEMF.

### ACKNOWLEDGMENTS

This work was supported in part by the Committee to Promote Research on the Possible Biological Effects of Electromagnetic Fields, Ministry of Internal Affairs and Communications.
